# Systemic Absorption of Catechins after Intraruminal or Intraduodenal Application of a Green Tea Extract in Cows

**DOI:** 10.1371/journal.pone.0159428

**Published:** 2016-07-18

**Authors:** Silvia Wein, Birgit Beyer, Annika Gohlke, Ralf Blank, Cornelia C. Metges, Siegfried Wolffram

**Affiliations:** 1 Institute of Animal Nutrition & Physiology, Christian-Albrechts-University of Kiel, Kiel, Germany; 2 Institute of Nutritional Physiology, Leibniz Institute for Farm Animal Biology, Dummerstorf, Germany; University of Alberta, CANADA

## Abstract

Green tea catechins have various potential health benefits in humans including anti-inflammatory, anti-oxidative and hepato-protective effects. If present in the circulation, they might have similar effects in ruminants, which are exposed to oxidative stress and fatty liver disease such as dairy cows during the periparturient phase. However, the bioavailability of a substance is a prerequisite for any post absorptive effect *in vivo*. This study aimed to investigate the appearance of catechins from a green tea extract (GTE) in cattle plasma after intraruminal and intraduodenal administration because absorption is of major importance regarding the bioavailability of catechins. The studies were performed in 5 rumen-fistulated non-lactating heifers and 6 duodenally fistulated lactating dairy cows, respectively, equipped with indwelling catheters placed in a jugular vein. The GTE was applied intraruminally (10 and 50 mg/kg BW, heifers) or duodenally (10, 20 and 30 mg/kg BW, dairy cows) in a cross‐over design with a 2 d washout period between different dosages. Blood samples were drawn following the GTE administration at various pre-defined time intervals. The concentration of the major GTE catechins (gallocatechin, epigallocatechin, catechin, epicatechin, epigallocatechin-gallate, epicatechin-gallate) in plasma samples were analysed by HPLC with electrochemical detection. Irrespective of the dose, almost none of the catechins originally contained in the GTE were detected in plasma samples after intraruminal application. In contrast, intraduodenal administration of GTE resulted in increased plasma concentrations of epicatechin, epigallocatechin, epigallocatechin gallate in a dose‐dependent manner. Thus, we can conclude that intraruminally or orally administered catechins are intensively metabolized by ruminal microorganisms.

## Introduction

**Fig 1:**
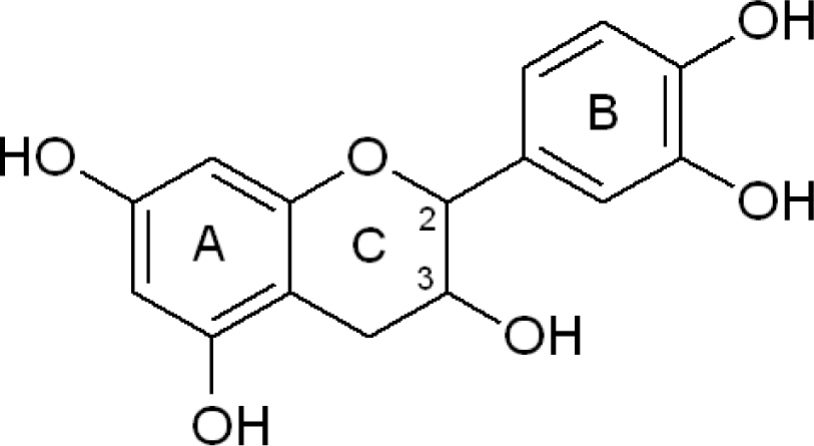
Basic flavanol skeleton. Catechin possesses two benzene rings (called the A- and B-rings) and a dihydropyran heterocycle (the C-ring) with a hydroxyl group on carbon 3. The molecule possesses two stereogenic centers on carbons 2 and 3.

Flavonoids are a large group of polyphenolic plant metabolites commonly present in higher plants [1]. According to their chemical structures, flavonoids are classified into six main subclasses: flavones, flavonols, flavanols (catechins), flavanones, isoflavones and anthocyanidins [2]. Differences between individual representatives within these groups consist mainly in the number and position of hydroxyl groups attached to the ring system (Fig 1). The subgroup of flavanols includes both monomeric catechins (e.g. catechin), and substances formed by polymerization/condensation (e.g. proanthocyanidine, theaflavine, thearubigene). Catechin (Fig 1) possesses two benzene rings (A- and B-rings) and a dihydropyran heterocycle (C-ring) hydroxylated at carbon 3 [3]. In contrast to most other flavonoids, which are present in the plant as glycosides, the flavanols occur mainly as aglyca or as gallic acid esters of the catechins [4].

**Fig 2:**
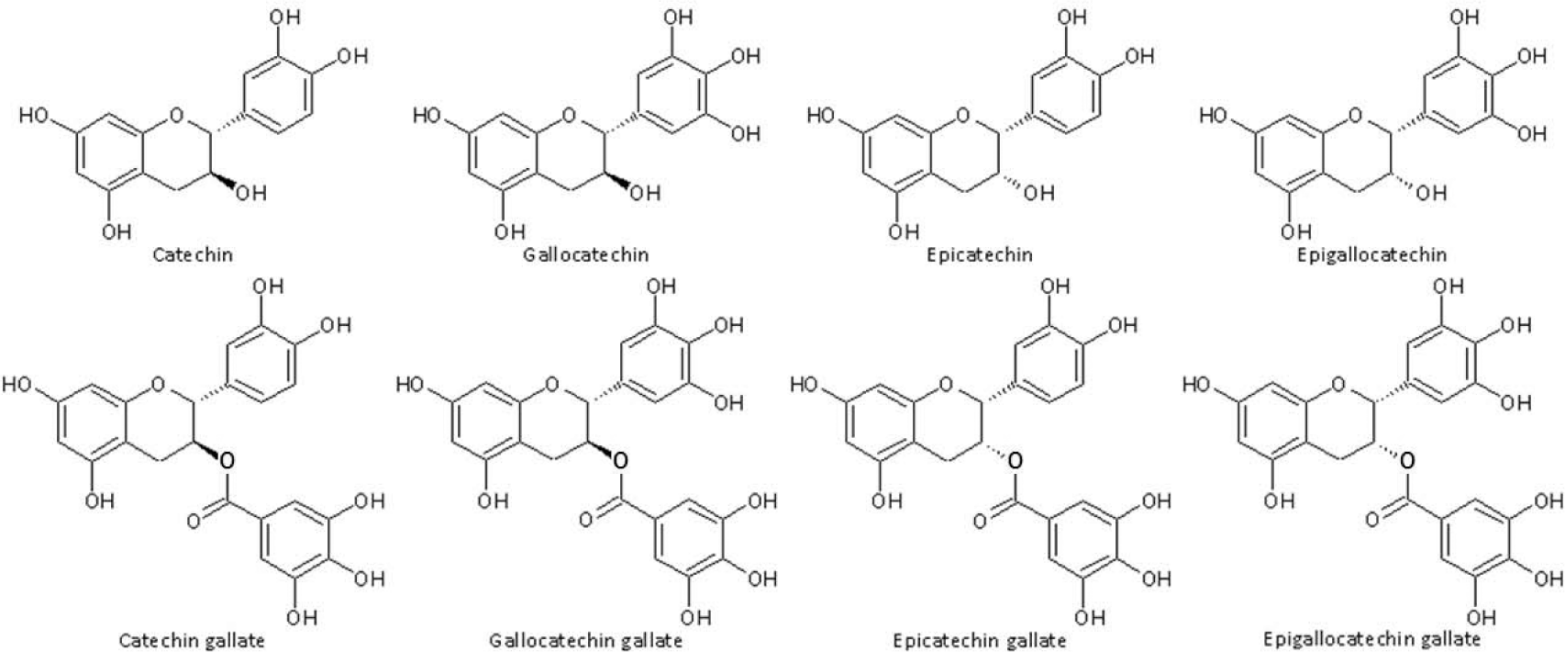
Structure of different green tea catechin derivatives.

Flavanols possess two chiral carbons (C2 and C3 of ring C), resulting in four diastereoisomers for each of them. Catechin and epicatechin are epimers, with (-)-epicatechin (EC) and (+)-catechin (C) being the most common optical isomers found in nature [5]. Presence of three hydroxyl groups at ring B of the aglycone leads to the formation of gallocatechins such as (-)-epigallocatechin (EGC) and (+)-gallocatechin (GC). Conjugation of hydroxyl groups on the pyran ring with gallic acids leads to ester formation such as (-)-epicatechin-3-gallate (ECG) and (-)-epigallocatechin-3-gallate (EGCG) (Fig 2). The most significant source of monomeric catechins is tea (*Camellia sinensis*), where they account for about one-third of the dry matter content of tea leaves [3]. The quantitatively most abundant catechin derivative in fresh tea leaves is EGCG [4,3,5].

Bioavailability of a substance is a prerequisite for any post absorptive effect *in vivo*. The oral bioavailability of a substance is pharmacologically defined as the extent and rate at which the active moiety (drug or metabolite) is present in the systemic circulation after oral application, thereby accessing the site of action. Thus, the bioavailability of a compound is usually determined by its concentration-time profile in systemic circulation and assessed by calculating the area under the plasma concentration time curve (AUC) [6]. The bioavailability and biotransformation of tea catechins in mammals has been already investigated in several studies. Experimental evidence indicates the involvement of simple passive diffusion as well as carrier-mediated facilitated diffusion in uptake of catechins across the brush-border membrane [7]. In addition, efflux transporters such as P-glycoprotein or members of the multidrug resistance protein family seem to be involved in the absorption and excretion of green tea catechins [8]. During the course of absorption in the small intestine and later in the liver, flavonoids undergo extensive methylation, glucuronidation, and sulfation [9]. However, there are considerable species- and tissue-specific differences among monogastric species in these reactions which are reviewed elsewhere [2]. In addition to these conjugation reactions, catechins present in the intestinal lumen undergo extensive microbial metabolism resulting in the ring fission products 5-(3,4,5-trihydroxyphenyl)-γ-valerolactone (M4), 5-(3,4-dihydroxyphenyl)-γ-valerolactone (M6) and 5-(3,5-dihydroxyphenyl)-γ-valerolactone (M6) [10]. These metabolic intermediates are further degraded by the gut flora to phenylacetic and phenylpropionoic acids [11]. After oral administration of green tea, EC, EGC and EGCG were detected in micromolar or submicromolar range in the blood samples of monogastric species including humans [12,13], mice [14,15], rats [16,17], and dogs [18,19]. Among all tea catechins, EGCG has been shown to be responsible for most of the health promoting effects of green tea [9]. Possible beneficial health effects of green tea are being extensively investigated and have received a great deal of attention in recent times [20,9,21,22]. EGCG has been shown *in vitro* and partially *in vivo* to possess strong anti-oxidant, anti-inflammatory, and anti-proliferative activities and thus potentially exhibits hepato-protective properties [23,24,25]. Although the majority of data on biological effects are derived from cell culture studies and experiments in monogastric species, it can be hypothesized that catechins, if present in the circulation, might have similar effects in ruminants.

Dairy cows are exposed to oxidative stressors during the periparturient phase and can suffer from metabolic disorders such as fatty liver disease and ketosis [26,27]. Thus, cows might benefit from the above mentioned health promoting properties of green tea. However, as far as ruminants are concerned, to date no systematic studies on the bioavailability of green tea catechins are available. At least in monogastric species, green tea catechins are mainly absorbed from the small intestine and it can be assumed that extensive microbial degradation might occur in the forestomachs. The objective of this study was to explore the net absorption of green tea catechins within the gastrointestinal tract of cows after intraruminal or intraduodenal administration, respectively.

## Materials and Methods

The study described here is comprised of two independent experiments: Experiment 1 was performed in non-lactating heifers equipped with a rumen fistula, whereas experiment 2 was conducted using lactating dairy cows equipped with a duodenal fistula. Whereas intraruminal application can yield information about the absorption of catechins after oral intake in ruminants (experiment 1), the experiments with intraduodenal application of green tea extract (GTE) provide insight about the absorption of catechins from the small and large intestine after circumvention of the forestomachs (experiment 2). Additionally, two *in vitro* experiments using the Hohenheim Gas Test (HGT) were performed to analyse microbial degradation of green tea catechins (experiment 3), and to identify possible effects of green tea catechins on microbial fermentation (experiment 4), respectively. For all experiments within the study, the same green tea extract (GTE, Polyphenon 60, Sigma Aldrich Chemie GmbH, Taufkirchen, Germany; composition Table 1) was used.

**Table 1:** Catechins of Polyphenon 60 extract from green tea^a^.

**Catechin derivatives**	**content [%]**
Epigallocatechin (EGC)	19.0
Epicatechin (EC)	6.4
Epigallocatechin gallate (EGCG)	28.8
Epicatechin gallate (ECG)	7.0
Gallocatechin gallate (GCG)	2.1
Catechin gallate (CG)	0.3
Gallocatechin (GC)	5.2
Catechin (C)	1.4

^a^ according to supplier’s declaration (Sigma Aldrich Chemie GmbH, Taufkirchen, Germany); also includes proteins (approx. 18%), caffeine, minerals and fiber.

### Experiment 1: Systemic absorption of catechins after intraruminal application

The study was performed in 5 rumen-fistulated non-lactating heifers (Deutsches Schwarzbuntes Niederungsrind, mean body weight (BW) 479 ± 15 kg (SEM) in a crossover design with a 2-d wash-out period between each application. Animals were surgically equipped with indwelling catheters placed in one jugular vein (VWI Jugularis Teflon catheter, C. Walter, Baruth/Mark, Germany) and were fed a diet consisting of 1.5 kg concentrate and 1.5 kg hay (dry matter, DM) twice daily with free access to tap water. The concentrate was composed of (% of DM) 23.2 rye, 21 rapeseed extraction meal, 18 corn gluten feed, 15 milled barley grain, 12 rye bran, 4.5 beet pulp, 4 corn, and 1.05 calcium carbonate, with the addition of 7,000 IU of vitamin A, 850 IU of vitamin D3, and 7.5 mg of copper (from copper-II-sulfate, pentahydrate) per kg of concentrate feed. Additionally, the cows received daily 75 g of mineral premix containing (%) 41.6 calcium carbonate, 24.4 sodium chloride, 13.9 calcium sodium phosphate, 7.2 magnesium oxide, 5 sugar cane molasses, and 2 mono-calcium phosphate.

Three days after catheter implantation to one jugular vein, the GTE, suspended in 500 mL of physiological saline, was administered using two dosages (10 and 50 mg GTE/kg of BW) via rumen fistula during morning feeding. Pure saline was used as control.

Blood samples (9 mL) were collected via jugular catheters into lithium-heparinized monovettes (Sarstedt, Nümbrecht, Germany) before (time point zero) as well as 0.5, 1, 1.5, 2, 2.5, 3, 4, 6, 8, 12, 24 and 36 h after intraruminal GTE application. Samples were immediately centrifuged (1,100 × g, 10 min, 4°C) and aliquots of plasma were mixed with a stabilization solution (20 μL/ mL plasma, 0.4 M NaH_2_PO_4_ with 20% ascorbic acid and 0.1% ethylenediaminetetraacetic acid, pH 3.6) to protect catechins from oxidation and degradation reactions [28,29]. Samples were stored at -80°C until analysis.

The experiment was approved by the Ministry of Agriculture, the Environment and Rural Areas of the state Schleswig-Holstein, Germany (permission no. V242-7224.121–25) and were in accordance with the guidelines issued by the German authorities for care and treatments of animals [30].

### Experiment 2: Systemic absorption of catechins after intraduodenal application

The study was performed in 6 duodenally fistulated [31] lactating dairy cows (German-Holstein, mean BW 598 ± 28 kg (SEM)) at 83–88 days in milk in their second lactation in a crossover design with a 2-d wash-out period between applications. Animals were surgically equipped with indwelling catheters placed in one jugular vein (Certofix mono, B. Braun Melsungen AG, Melsungen, Germany) and were fed a TMR for *ad libitum* intake and had free access to tap water. Diet composition was calculated for lactating cows according to the recommendations of the German Society of Nutritional Physiology [32]. The TMR was composed of (g/kg of DM) 199 grass silage, 317.3 corn silage, 19.7 barley straw, 19 hay, 73.5 molassed sugar beet pulp (Arp, Thordsen, Rautenberg GmbH & Co KG, Sollerupmühle, Germany: 7.3 MJ of NEL/kg of DM, 153 g of utilizable protein/kg of DM), 11.6 Canola meal extract, 342.1 concentrate (MF 2000, Vollkraft Mischfutterwerke GmbH, Güstrow, Germany: 33% extracted soy meal, 20% corn, 17% wheat gluten, 8% extracted rapeseed meal, 5% sugar beet pulp, 2% sodium hydrogen carbonate, 1.3% calcium carbonate, 0.2% sodium chloride, 8.0 MJ of NEL/kg of DM, 204 g of utilizable protein/kg of DM), 9.7 propylene glycol (Propylene glycol USP, Dr. Pieper Technologie- und Produktentwicklung GmbH, Wuthenow, Germany), 8.5 supplemented minerals (Rinderstolz 9522 lactation, Salvana Tiernahrung GmbH, Sparrieshoop, Germany: 92% crude ash, 20% calcium, 5% phosphorus, 6% magnesium, 8% sodium, vitamins). Cows were milked twice daily at 0400 and 1430 h.

Three days after vein catheter implantation, GTE was administered in NaCl solution into the duodenum at 3 different dosages (10, 20, 30 mg GTE/kg BW). Pure saline was used as control. Blood samples (9 mL) were collected via jugular catheters into lithium-heparinized monovettes (Sarstedt, Nümbrecht, Germany) two times before (0.5 and 0.25 h) as well as 0.5, 1, 1.5, 2, 2.5, 3, 4, 6, 8, 12, 24 and 36 h after intraduodenal GTE application and were treated as described in experiment 1.

The experimental procedures were carried out in accordance with the German Animal Protection regulations and were approved by the relevant authorities of the State Mecklenburg-Vorpommern, Germany (Landesamt fur Landwirtschaft, Lebensmittelsicherheit und Fischereiwesen Mecklenburg-Vorpommern, Germany; permission no. 7221.3–1.1-063/10).

### Experiment 3: Ruminal degradation of catechins *in vitro*

Ruminal degradation of catechins was assessed *in vitro* using the HGT according to the method of Menke and Steingass 1988 [33]. The preparation of incubation medium (IM) and the incubation experiments were performed according to a standard procedure [34]. For the experiment, a substrate consisting of grass hay and concentrate (50:50, w:w fresh weight) was supplemented with 160 mg GTE, resulting in final concentrations of the individual catechins GC, EGC, EC and EGCG of 27, 100, 35, and 100 (μmol/L). The control consisted of unsupplemented substrate (control). Two-hundred mg of the supplemented substrate was incubated with rumen fluid in calibrated glass syringes in 30 mL of IM. Rumen fluid was collected under anaerobic conditions from two rumen fistulated heifers used in experiment 1, which were fed as described. After collecting and filtering through a finely woven cloth into a thermos bottle, rumen fluid was immediately mixed with a freshly prepared buffer solution [34]. Glass syringes were filled and subsequently placed in a rotator-consisting incubator held at 39°C. Samples were removed from the incubator after 0, 0.5, 1, 1.5, 2, 2.5, 3, 4, 6, 8, 12, and 24 h and transferred into ice-cooled tubes to stop further microbial degradation. To determine a spontaneous decay of catechins not caused by microbial activities, four additional runs with 160 mg GTE/L were performed using inactivated IM (boiled for 20 min to stop bacterial activities). All samples were stored at -80°C until analysis.

### Experiment 4: Impact of catechins on gas production *in vitro*

Effect of catechins on total gas production was determined using the HGT [33]. The experimental procedures, feeding of donor animals and preparation of the IM were performed as described in experiment 3. In this experiment, however, a mixture of concentrate and grass hay (50:50) supplemented with 20 or 40 mg GTE/L IM, respectively, was used as substrate. Additionally, three runs containing only IM (without feed samples) were performed within each batch as blind samples to evaluate intrinsic gas production. Simultaneously, two reference standards (concentrate and grass hay) obtained from the University of Hohenheim (Stuttgart, Germany) were incubated in each run. The mean correction factor was set to be in the range between 0.9 and 1.1, elsewise the run was repeated. Cumulative gas production was measured over 24 h.

### Catechin analysis

The concentration of the major catechins in samples of plasma (GC, EGC, C, EC, EGCG, ECG) and of ruminal fluid (EC, EGC, EGCG, GC) were analysed by HPLC with electrochemical detection according to the method described previously [13]. Briefly, 500 μL thawed plasma was incubated for 45 min at 37°C with an enzyme mixture (ß-glucuronidase with sulphatase activity, type H-1 from *helix pomatia*, final activities 7,300/130 U/mL glucuronidase/sulphatase) for cleavage of all ester bonds of glucuronated and sulfated catechins. Subsequently enzymatic cleavage was stopped by adding 1 mL methylene chloride to the sample. After shaking and centrifuging (15 min at 2,000 × g and 4°C) the supernatant (400 μL) was transferred to test tubes and mixed with 1 mL ethyl acetate. Then, the sample was mixed and centrifuged (20 min, 2,000 × g, 4°C) again. The organic phase (800 μL) was transferred to a glass test tube, and the ethyl acetate extraction was repeated twice. The thus obtained three supernatants were pooled, mixed with 10 μL of 1% aqueous ascorbic acid and evaporated until dryness (partial vacuum at 45°C, Savant Speed Vac^®^ Systems AES 1010, Global Medical Instrumentation, USA). The dried sample was resolved in 150 μL of mobile phase A (see below) and after mixing and sonication 30 μL of the resulting supernatant was injected into the HPLC. Samples of ruminal fluid were thawed, centrifuged (15 min, 2,000 × g, 4°C) and refrigerated again for at least 24 h prior to the extraction, performed as in plasma samples. It should be mentioned here, that the fact, that we did not use an internal standard during the analyses of catechins is critical for a precise quantitative analysis. The main goal of the present study was, however, to get principal information, whether catechins are or are not absorbed in cows.

Analysis was performed on a JASCO 2000Plus HPLC-system (pump PU-2080Plus, low pressure gradient unit LG-2080, degasser DG-2080.54, autosampler AS-2057Plus, column oven Jetstream II Plus; JASCO Germany GmbH, Gross-Umstadt, Germany) linked with a 4-channel coulometric electrochemical detector (CoulArray; ESA Inc., Massachusetts, USA). For separation the sample passed through a reversed-phase column (C-18 Kromasil 100, 25 × 4.6 mm, particle size 5 μm; JASCO) guarded by a pre-column (C-18 Inertsil ODS 2, 10 × 4 mm, particle size 5 μm; JASCO) placed in a column oven at 30°C. Mobile phase A and B (pH 2.5) consisted of ultrapure water, acetonitrile, and trifluoracetic acid at the ratio of 92:8:0.1 and 65:35:0.1 (v/v/v), respectively. The flow rate came to 0.9 mL/min and the eluent was monitored at electrochemical potentials of 0, 120, 240, and 360 mV. To quantify single catechins, channels with dominant signals were used, respectively (EGC and ECG at 120 mV, GC, EGC, C and EC at 240 mV). The detection limit was 0.01 μmol/L and inter-analysis and inter-day variances were within 5% for all catechins analysed. For quantification of individual catechins in plasma, external standards were used (Carl Roth GmbH & Co. KG, Karlsruhe, Germany). Standard curves were generated by adding EGC, ECG, EC, C, EGCG, and GC to blank plasma at final concentrations of 10, 7.5, 5, 2.5, 1, 0.5, 0.25 and 0.125 μmol/L. Standards were treated the same way as experimental plasma samples. Identification of catechins in plasma samples was performed using the retention times of pure catechins (standards). The standard curves displayed linearity for all catechins with r ≥ 0.99.

### Pharmacokinetic calculation and statistics

All data presented are mean values ± SEM. In experiment 2 pharmacokinetic parameters (C_max_ = maximum plasma concentration, t_max_ = time to achieve maximum plasma concentration, AUC = area under the curve) were calculated for each dose (10, 20 and 30 mg / kg BW) regarding total plasma catechins (calculated as the sum of the concentrations of C + EC + EGC + EGCG + ECG + GC). Furthermore, values shown always represent the sum of conjugated and non-conjugated forms, because all samples were enzymatically with ß-glucuronidase/sulfatase. AUCs were calculated according to the trapezoidal rule for the period between 0 and 36 h. Statistical differences were calculated using GLM and PROC MIXED of SAS. For statistical evaluation of experiments 3 and 4 a two-factorial ANOVA was performed followed by Bonferroni post hoc test using SAS (SAS Institute, Inc. Version 9.2). In all experiments a P-value ≤ 0.05 was considered to be significant.

## Results

### Experiment 1: Systemic absorption of catechins after intraruminal application

**Fig 3:**
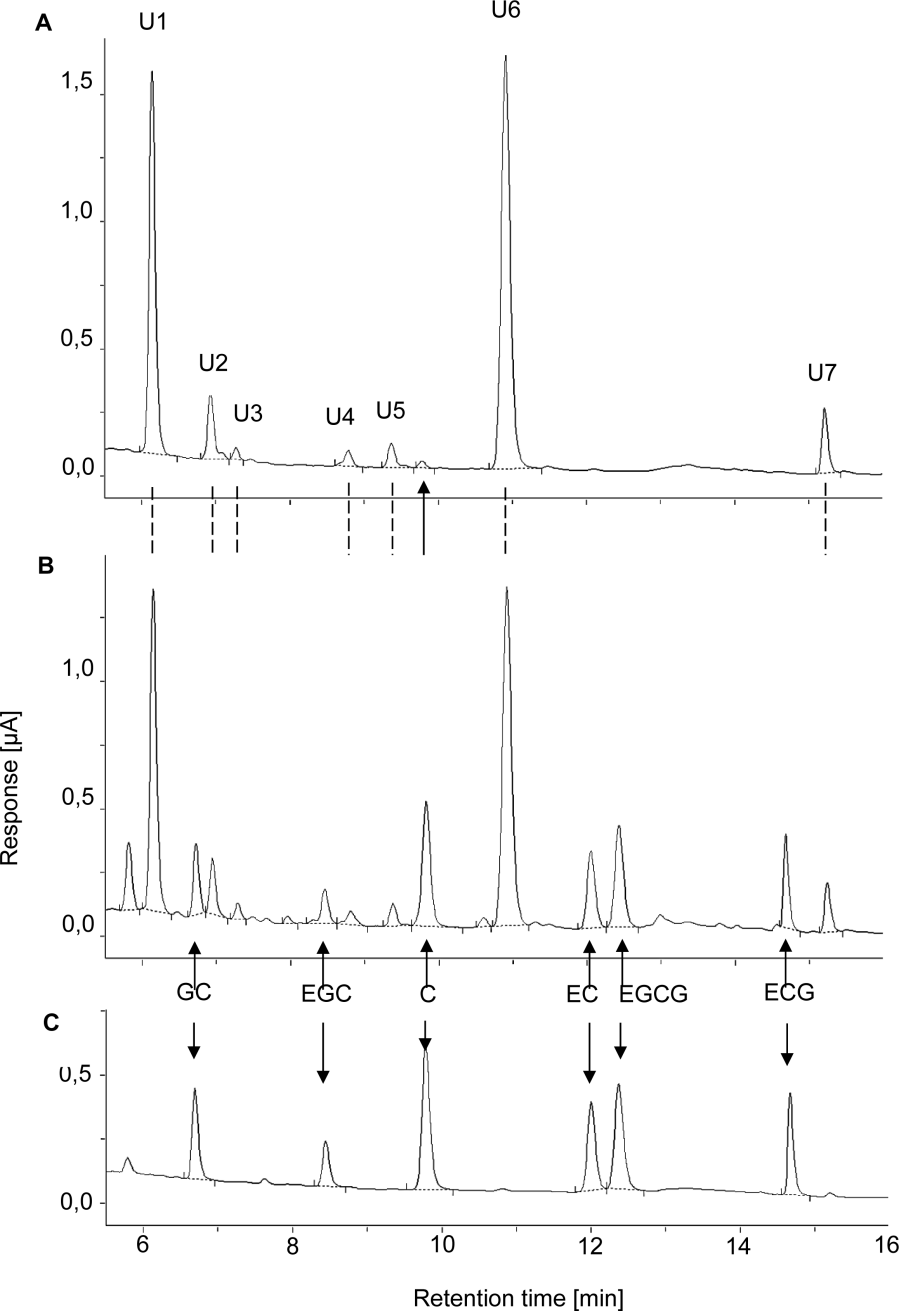
**Representative HPLC chromatograms of a plasma sample obtained 2.5 h after intraruminal administration of 50 mg/kg BW Polyphenon 60 (A), the same sample spiked with standard (1 μmol of each individual catechin/L plasma) (B), and the pure standard solutions (C).** U = unknown, C = catechin, EC = epicatechin, ECG = epicatechin-3-gallate, EGC = epigallocatechin, EGCG = epigallocatechin-3-gallate, GC = gallocatechin.

In the first experiment plasma total catechin concentrations were analysed after intraruminal administration of GTE at a dose of 10 and 50 mg per kg of BW, respectively. Regardless of the dose, almost none of the original substances present in the GTE could be detected in plasma with the exception of catechin, which appeared in very low concentrations (Fig 3). However, seven unidentified peaks (U1-U7) most likely presenting metabolites of the catechins administered were detected in plasma samples (Fig 3).

### Experiment 2: Systemic absorption of catechins after intraduodenal application

**Fig 4:**
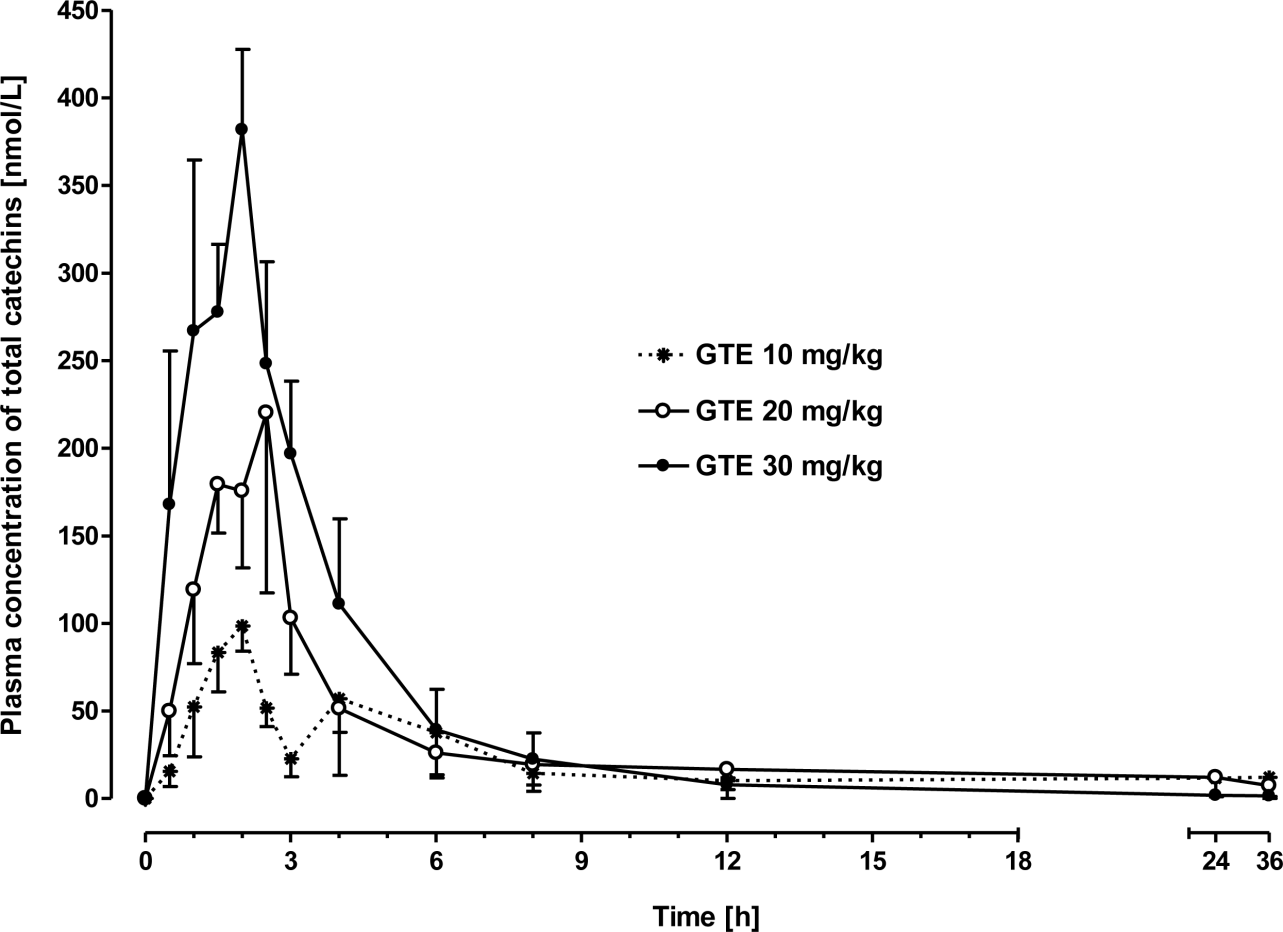
Plasma concentration-time curves of total catechins (TC) as the sum of conjugated and non-conjugated forms after intraduodenal application of green tea extract (GTE) at doses of 10, 20 or 30 mg/kg BW, respectively. Total plasma catechins were calculated according to: TC [nmol/L] = C + EC + EGC + EGCG + ECG + GC. Values are means ± SEM of 6 lactating cows.

**Fig 5:**
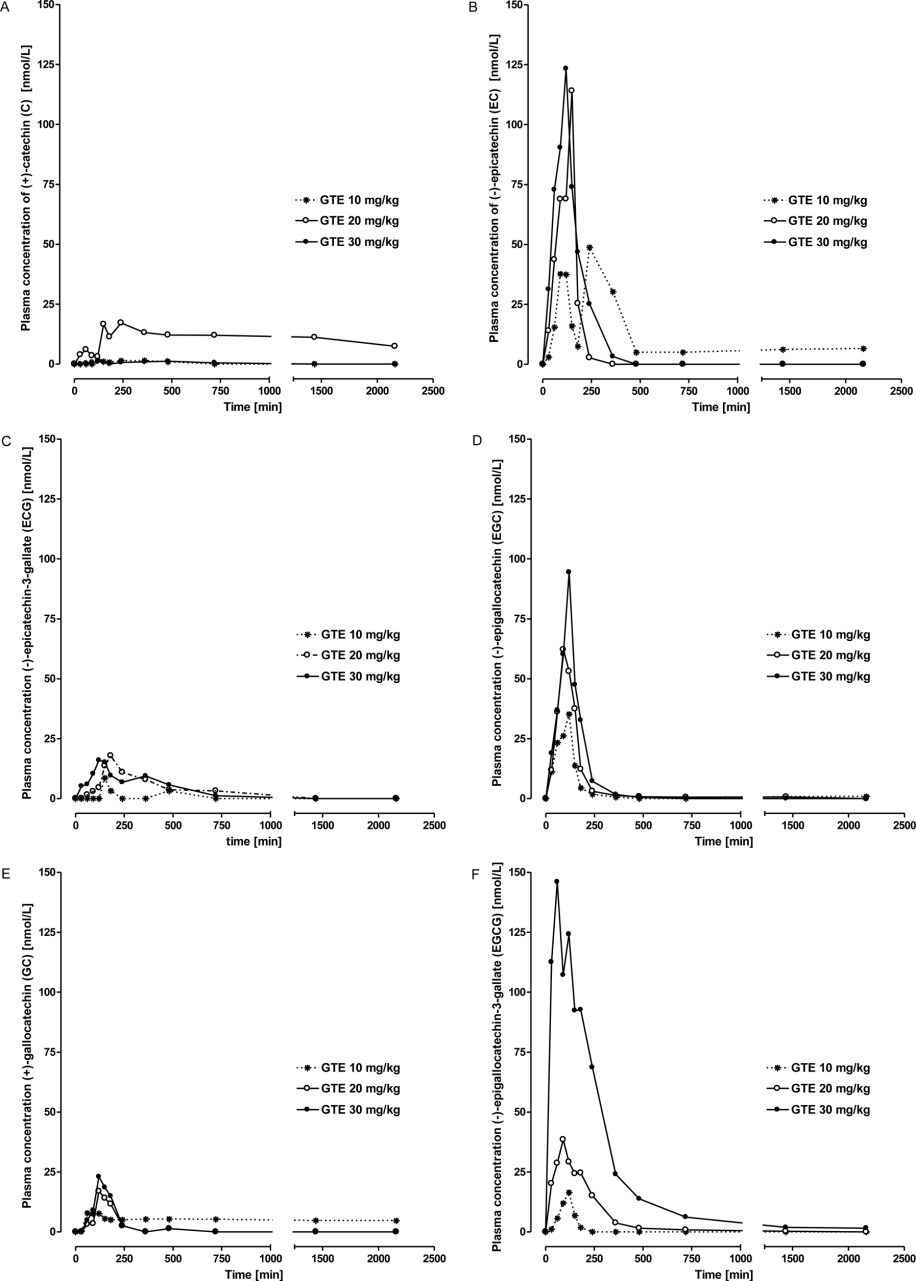
**Plasma concentration-time curves of catechin derivatives as the sum of conjugated and non-conjugated forms (A-F; C, EC, ECG, EGC, GC, EGCG) after intraduodenal application of green tea extract (GTE) at a dose of 10, 20 or 30 mg/kg BW, respectively.** Values are means of 6 lactating cows (for the sake of clarity, SEM are not depicted).

After intraduodenal catechin administration plasma concentrations of total catechins (TC) (Fig 4) as well as of the individual catechins EC, EGC, EGCG, clearly increased above baseline in a dose-dependent manner (P < 0.0001), while plasma C, ECG, and GC values did not differ from baseline irrespective of the dose administered (P > 0.5) (Fig 5A–5F). Plasma concentrations of TC, EC, EGC, and EGCG peaked (P < 0.01) between 1.5 and 2.5 h after administration of 20 and 30 mg/kg BW catechins (P < 0.05) and returned to baseline values between 2.5 and 4 h after administration, respectively (Figs 4 and 5A–5F, Table 2). In the baseline samples no catechins were detected and after administration of 10 mg/kg BW, plasma values were not different from baseline (P > 0.5).

**Table 2:** Plasma kinetic parameters of total catechins after intraduodenal bolus application of a green tea extract (GTE).

	10 mg GTE	20 mg GTE	30 mg GTE
Total plasma catechins^a^^,^^b^			
C_max_ (nmol/L)	98 ± 14	220 ± 102	382 ± 45
T_max_ (min)	125 ± 30	110 ± 13	105 ± 10
AUC _0–36_ (μmol • min^-1^ • L^-1^)	39.9 ± 22	57.6 ± 23	73.6 ± 18

AUC = area under the plasma concentration–time curve, C_max_ = the maximum plasma concentration, and T_max_ = the time to reach the maximum concentration

^a^ Values are means ± SEM; n = 6

^b^ Total plasma catechins were calculated according to: total catechins (nmol/L) = C + EC + EGC + EGCG + ECG + GC

### Experiment 3: Ruminal degradation of catechins *in vitro*

**Fig 6:**
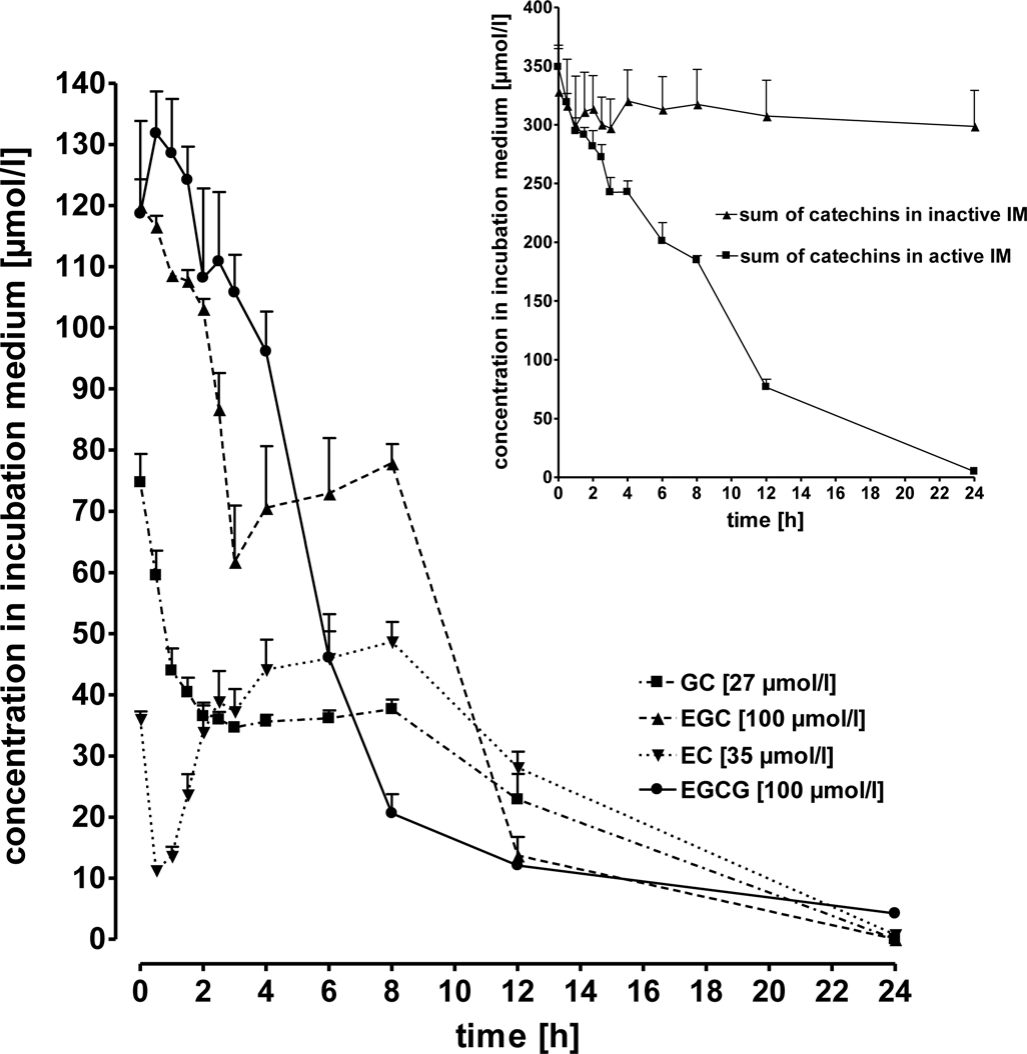
Concentration-time curves of GC, EGC, EC and EGCG (initial concentrations, μmol/L: 27, 100, 35, and 100, respectively) in the Hohenheimer Gas Test incubation medium over 24 h. Values are means + SEM of 5 observations. Inset: concentration-time curves of the sum of catechins (GC, EGC, EC, EGCG) using either active or inactive incubation medium (IM).

Concentration-time curves of GC, EGC, EC and EGCG (initial concentrations, μmol/L: 27, 100, 35, and 100, respectively) were measured in the incubation medium over a period of 24 h. During the incubation period the concentrations of the catechins investigated decreased continuously. After 24 h concentrations were below the detection limit (Fig 6). Microbial degradation of catechins was confirmed by measuring the concentrations of total catechins using active versus inactivated rumen fluid (Fig 6, inset). Whereas the use of active rumen fluid resulted in a time-dependent decrease of total catechin concentrations, the initial catechin concentration remained more or less constant when inactivated ruminal fluid was used for incubation (Fig 6, inset).

### Experiment 4: Impact of catechins on gas production *in vitro*

**Fig 7:**
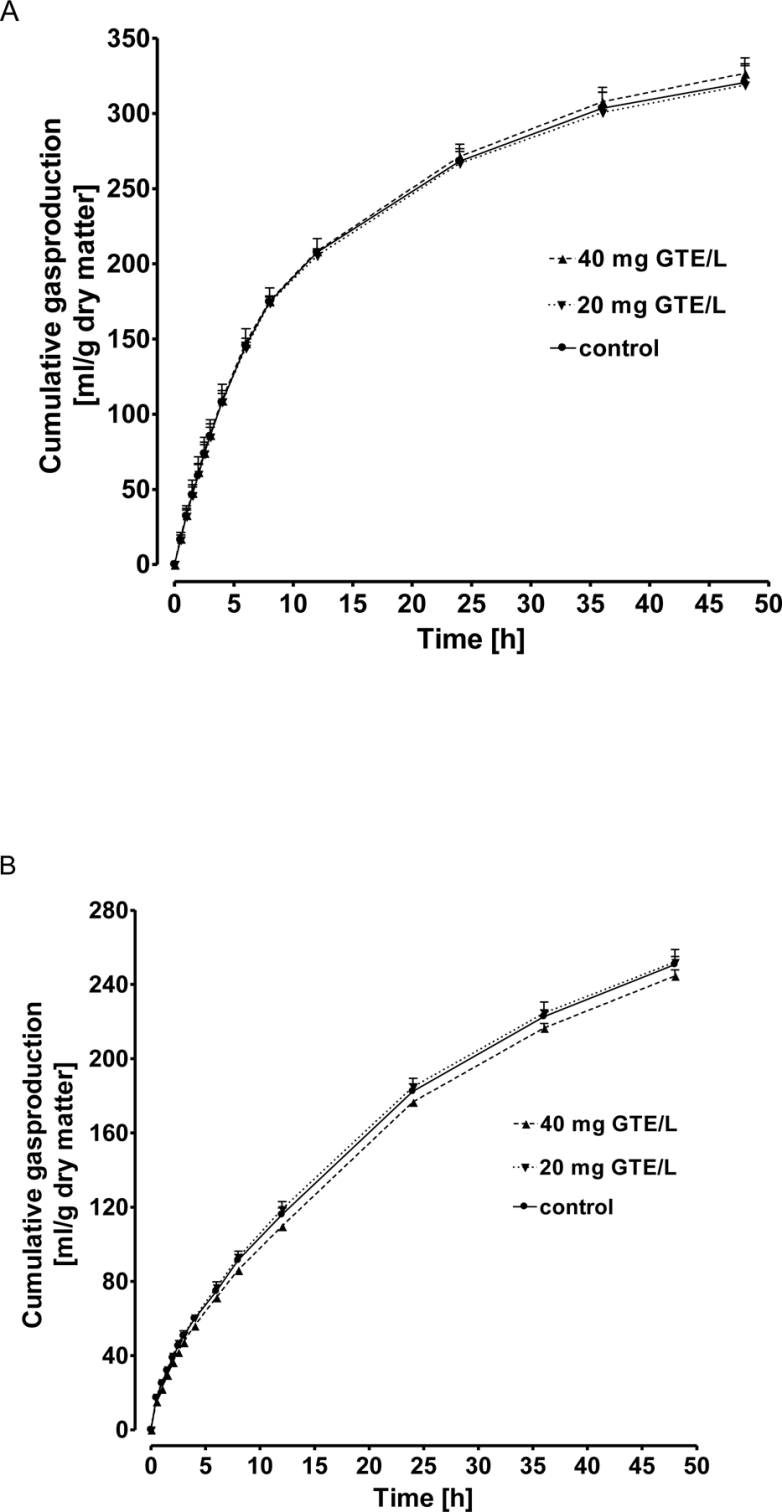
**Impact of a green tea extract (GTE 20 or 40 mg/L respectively) on in vitro gas production using either concentrate (A) or grass hay (B) as substrates.** Controls were run without the addition of GTE. Values are means + SEM, of 4 (A) or 3 (B) observations, respectively.

The impact of GTE (20 or 40 mg/L respectively) on *in vitro* gas production was investigated over an incubation period of 48 h using either concentrate or grass hay as substrate. Controls were run without the addition of GTE. Total gas production did not differ (P > 0.5) irrespective of GTE supplementation and dose used (Fig 7).

## Discussion

Flavonoids are a large, heterogeneous group of phytochemicals with polyphenolic structure that are present in variable concentrations in almost all higher plants. Numerous *in vitro* and *in vivo* experiments in monogastric species (human, rat, mouse) have shown that catechins, which are among the most common flavonoids in many higher plants, have antioxidant, antifungal, antibacterial, and anti-inflammatory properties [8]. In addition, the expression of numerous genes and the activity of key enzymes and metabolic processes are affected by catechins [35]. In particular, during early lactation dairy cows experience an enormous physiological adaptation, resulting in massive mobilization of body fat and protein reserves. This is often associated with health problems such as fatty liver, increased lipid peroxidation, or ketoacidosis. Pathophysiological changes include increased plasma concentrations of non-esterified fatty acids, ß-hydroxybutyrate, oxidatively modified lipids and hepatic lipid accumulation [36,37]. It is also known that the antioxidant capacity in high-yielding dairy cows is affected in the periparturient period [38]. Although positive health effects of polyphenols, including catechins, have been demonstrated in humans [4,20] and monogastric species [39,40] and has been partly shown and postulated also in ruminants [41,42] appropriate investigations in ruminants are not available. Thus, we investigated for the first time the systemic appearance of catechins contained in a green tea extract after intraruminal and intraduodenal application, respectively.

Our results clearly demonstrate the impact of ruminal microbiota on the degradation of catechins and the negative consequences for their systemic bioavailability. Similar to the processes in the large intestine of monogastric species, where extensive microbial degradation of catechins into phenolic acids has been shown [43,8,44,45,46], we here report rapid microbial degradation of catechins in the ruminal fluid. The role of rumen microbes could be clearly demonstrated because inactivated rumen fluid does not result in a substantial degradation of catechins. Microbial degradation of catechins in the forestomaches is most likely the principal reason for the lack of systemic availability of catechins after intraruminal application, and thus, oral intake. After intraruminal application no detectable plasma concentrations of any catechin (C, EC, ECG, EGC, GC) originally present in the GTE nor of glucuronidated, sulfated or methylated derivatives thereof were found. However, HPLC analysis revealed several unknown peaks which are most likely degradation products of the catechins contained in the GTE, because those unknown peaks did not occur in chromatograms obtained from plasma of control animals obtaining only saline solution. Ruminal degradation of catechins is further substantiated by the findings obtained after intraduodenal application of the GTE in dairy cows. Intraduodenal GTE application of at least 20 mg/kg BW resulted in increases of EC, EGC, EGCG concentrations in blood plasma, whereas C, ECG and GC were not increased over baseline values. The increase of the dose from 20 to 30 mg/kg BW resulted in higher plasma concentrations of GTE constituents, in a dose dependent- manner.

Concerning possible health promoting effects of green tea catechins it seems that EGCG is of particular interest [9]. A recent *in vitro* study investigating anti-inflammatory effects found, that the half maximal inhibitory concentration (IC_50_) for EGCG at which 50% inhibition of cyclooxygenase (COX-1, COX-2) and lipoxygenase (1-LOX) activity occur, are 14.9, 10.7 and 5.9 μg/mL, respectively [47]. In our experiments the maximum plasma concentration of EGCG after the highest dose of GTE (30 mg/kg BW) applied intraduodenally in dairy cows was 146 nmol/L which is equivalent to 0.0669 μg/mL. Although we cannot comment on possible health benefits on the basis of the present study, *in vitro* and *in vivo* data suggest that higher EGCG plasma concentrations are necessary to realize possible health effects. Another study in rats investigating long term (108 weeks) effects of EGCG indicates reduced inflammation, and oxidative stress as well as liver protection at a dose of 25 mg EGCG/kg BW and day whereas 5 mg EGCG/kg BW and day were ineffective [48]. Aside from systemic effects, local effects of GTE, especially within tissues of the gastro-intestinal tract have to be considered due to the fact, that local concentrations of flavonoids within the alimentary tract after oral intake might be considerably higher than concentrations measured in the systemic circulation. Local effects might include mainly effects on enzyme activities, intestinal epithelial protection (cell differentiation/barrier function improvement) or the inhibition of intestinal pathogens such as *Clostridium difficile* (*C*. *difficile*). However, when considering local effects of GTE one must keep in mind that available data stem from studies in monogastric species or cell culture experiments. In this context different flavonoids including EGCG have been shown to inhibit pancreatic amylase activity [49,50,51] and thus are suggestive to modulate amylase-mediated starch digestion and following postprandial plasma glucose concentrations. While EGCG intake in wistar rats prior to intestinal ischemia reperfusion injury alleviated the pathological changes of the intestine probably by activation of PI3K/Akt signalling pathway [52] no protective effects of green tea extracts on the intestinal mucosa were found in rats exposed to Cd or Pb over a period of 12 weeks [53]. In addition, EGCG might inhibit the expression of virulence genes in *C*. *difficile* and thus might exert bactericidal activities in *C*. *difficile*-infected mice [54]. Concerning hepatic effects of green tea catechins after oral application in adult ruminants i.e. after rumen-gastroduodenal digestion, no sound data are available today.

The aim of the present study was to get information on the fate of catechins in cows after oral application. The strength of the present paper is that we provide evidence, that catechins are substantially degraded by rumen microorganisms resulting in no detectable plasma catechin concentrations. To this end, a limitation of the present study is that we have not identified possible bacterial catechin metabolites, neither in ruminal fluid nor in plasma samples. Another important result was that after circumventing the forestomaches by intraduodenal application of catechins, plasma concentration time profiles for catechins were principally similar to data published for monogastric species. Finally, it must be pointed out, that we only determined the relative bioavailability of catechins in cows after intraruminal and intraduodenal application, respectively, but not the absolute bioavailability. We conclude that further studies with oral or intraruminal application using catechins protected against microbial degradation could be valuable to test potential health promoting effects of green tea catechins in cattle. Unless such applications will be developed, neither the application of pure catechins nor of catechin containing supplements appears to be promising.

## Supporting Information

S1 Appendix Cow individual plasma concentrations of catechins [nmol/L]. (XLS)
